# The Use of Upper Extremity Tourniquets in Hand Surgery—Does Tourniquet Location Make a Difference? A Scoping Review

**DOI:** 10.1177/22925503251392574

**Published:** 2025-12-09

**Authors:** Kimberley Yuen, Laryssa Kemp, Isabella Churchill, Jessica Gormley, Cameron Leveille, Helene Retrouvey, Matthew McRae, Ammara Ghumman

**Affiliations:** 1 Division of Plastic Surgery, 3710 McMaster University, Hamilton, Ontario, Canada; 2 Queen's University School of Medicine, 104820 Queen's University, Kingston, Ontario, Canada; 3 Division of Plastic, Reconstructive, and Aesthetic Surgery, 7938 University of Toronto, Toronto, Ontario, Canada

**Keywords:** tourniquet, hand surgery, wrist, forearm, upper arm, tourniquet, chirurgie de la main, poignet, avant-bras, bras

## Abstract

**Introduction:** Arm and forearm tourniquets are routinely used in hand surgery to provide a “bloodless” operating field. Though not yet widely adopted, wrist tourniquets have been reported to be a more comfortable option compared to other tourniquet locations. Thus, the primary purpose of this study was to review the use of upper extremity tourniquets in awake hand surgery and report any tourniquet-related outcomes. **Methods:** Following PRISMA-ScR guidelines, Medline and Embase were searched for primary studies published from inception to August 22, 2024, reporting on the use of upper extremity tourniquets in adult patients undergoing hand surgery with local anesthetic. Tourniquet time and tourniquet tolerance, represented by visual analog scale (VAS) scores, were the main extracted outcomes. **Results:** Two hundred of 1528 studies were reviewed at the full-text level by 2 reviewers, and 26 studies were included. Four studies incldued both forearm and upper arm tourniquets and were treated as individual studies. Of these, there were 19 studies that used an upper arm tourniquet (n = 931 procedures), 9 studies that used a forearm tourniquet (n = 657 procedures), and 2 studies that used a wrist tourniquet (n = 130 procedures). Average tourniquet time for upper arm, forearm, and wrist tourniquets was 11.4, 11.6, and 16.5 min, respectively, and mean VAS score for tourniquet pain were 4.0, 3.3, and 0.4, respectively. **Conclusions:** We reviewed the use of upper extremity tourniquets for hand surgery and associated outcomes. While limited, the literature suggests that wrist tourniquets are the most comfortable tourniquet location and are well tolerated by both patients and surgeons. Future comparative clinical studies are required to better assess such outcomes.

## Résumé

**Introduction :** Les tourniquets du bras et de l’avant-bras sont utilisés systématiquement lors des chirurgies de la main pour obtenir un champ opératoire «sans saignement». Bien qu’ils ne soient pas généralisés, les tourniquets du poignet constitueraient une solution plus agréable que ceux utilisés à d’autres emplacements. L’objectif primaire de la présente étude visait à examiner l’utilisation des tourniquets des extrémités supérieures lors des chirurgies éveillées de la main et à déclarer les résultats liés aux tourniquets. **Méthodologie :** Conformément aux directives du PRISMA-ScR, les auteurs ont fouillé les bases de données Medline et Embase pour en extraire les études primaires publiées entre la création de ces bases de données et le 22 août 2024 sur l’utilisation des tourniquets des extrémités supérieures chez des patients adultes qui avaient subi une chirurgie de la main sous anesthésie locale. La durée d’utilisation du tourniquet et la tolérance à son utilisation, représentées par les scores de l’échelle analogue visuelle (ÉAV), étaient les principaux résultats extraits. **Résultats :** Au total, les chercheurs ont analysé le texte intégral de 200 des 1 528 études et en ont retenu 26. De ce nombre, 19 faisaient appel à un tourniquet du bras (n = 931 interventions), neuf, à un tourniquet de l’avant-bras (n = 657 interventions) et deux, à un tourniquet du poignet (n = 130 interventions). La durée d’utilisation moyenne du tourniquet du bras, de l’avant-bras et du poignet était de 11,4, 11,6 et 16,5 min, respectivement, et le score de l’ÉAV moyenne de la douleur causée par le tourniquet, de 4,0, 3,3 et 0,4, respectivement. **Conclusions :** Les chercheurs ont examiné l’utilisation des tourniquets des extrémités supérieures pour la chirurgie de la main ainsi que les résultats qui s’y rapportent. Bien que les données soient limitées, les publications indiquent que le tourniquet du poignet est l’emplacement le plus agréable et qu’il est bien toléré, à la fois par les patients et les chirurgiens. De futures études cliniques comparatives devront être réalisées pour mieux évaluer les résultats.

## Introduction

An upper extremity tourniquet has historically been employed during hand surgery to provide a “bloodless” operating field for the surgeon to visualize anatomical structures and perform precise interventions.^1^ The pneumatic tourniquet is most commonly used and consists of an inflatable cuff connected to a pressure-regulating device. Tourniquet pressure is typically inflated to 250 or 100 mm Hg above the patient's systolic blood pressure. This allows for precise pressure control, individualization to the patient's limb size, and quick deflation. While the tourniquet helps to provide hemostasis, tourniquet time must be monitored to prevent complications such as nerve and muscle ischemia.

In hand surgery, the tourniquet is commonly applied to the upper arm or forearm, depending on the location of the operative site and surgeon preference. An upper arm tourniquet is placed around the midhumerus proximal to the elbow. Historically, this was the placement of choice as it was thought to be safer to apply a tourniquet over an area that was well padded with muscle.^2^ However, it was soon discovered that the upper arm tourniquet induces more ischemic injury to muscular and neurovascular structures, increases pain and discomfort, and has the potential to inflict nerve injury, including both friction and chemical burns.^3,4^ Forearm tourniquets are typically placed distal to the antecubital fossa and are thought not to induce upper arm nerve compression, as this has clinically corresponded with a later onset of nerve paresthesia and muscle paralysis.^5^ However, some surgeons have expressed difficulty with forearm tourniquet placement for certain hand and wrist procedures due to decreased surgical field, as well as difficulty with finger positioning due to tourniquet pressure on the extrinsic flexors.^6,7^ Conflicting findings have been published comparing upper arm and forearm placements for tourniquet-related outcome measures, with some reporting less pain experienced with forearm tourniquets, and others reporting no change.^2,7–9^

To minimize the number of structures exposed to ischemia, wrist tourniquets are a third option for hand surgery. A wrist (or distal forearm) tourniquet commonly refers to a tourniquet placed 1 to 2 finger breadths proximal to the volar wrist crease.^8^ In 1990, Guirguis et al occluded flow to the hand by wrapping a surgical glove 3 times around the wrist.^10^ This achieved a bloodless surgical field in 96% (n = 24/25) of their procedures and was tolerated by all but 2 of the patients.^10^ While this study utilized both novel tourniquet placement and material, it did raise concerns regarding maintenance of variable pressures and skin injuries. Despite the introduction of wrist tourniquets for hand surgery over 30 years ago, wrist tourniquets have not been widely adopted.^10,11^ Thus, the primary purpose of this study was to review the literature on the use of upper arm tourniquets in awake hand surgery with a specific interest in the use of wrist tourniquets and report any tourniquet-related outcomes to date.

## Methods

### Search Strategy

**Figure 1.**
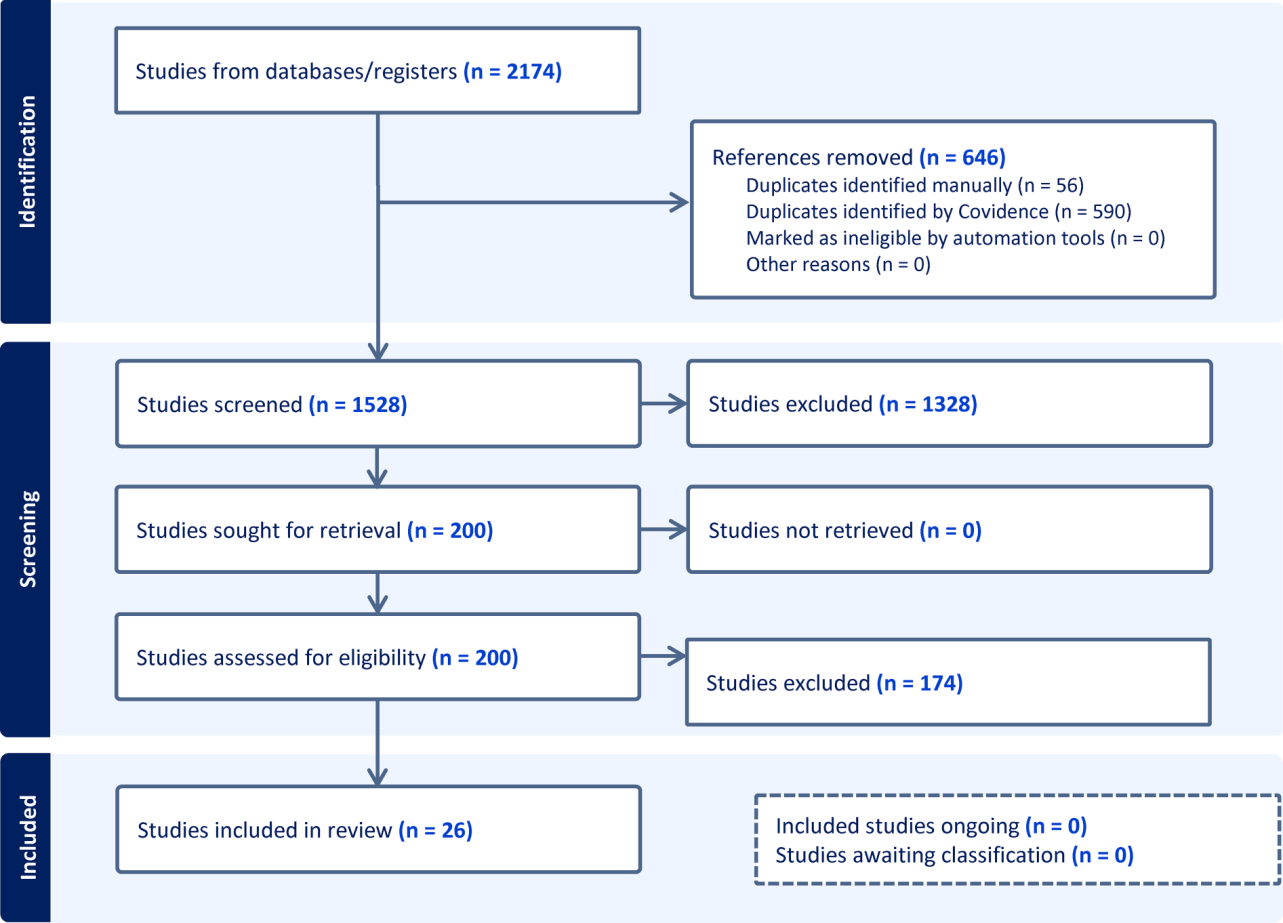
PRISMA ScR protocol schematic.

This study followed the Preferred Reporting Items for Systematic reviews and Meta-Analyses extension for Scoping Reviews (PRISMA-ScR) Checklist. The search strategy was developed in conjunction with a medical librarian and was a broad search including primary studies published from inception to August 7, 2023, reporting on the use of upper extremity tourniquets in adult patients undergoing hand surgery using local or regional anesthetic. Two databases were searched: Ovid Medline (1946 to August 2023) and Embase (1974 to August 2023). The search was then updated from the previous search end date (August 7, 2023) to August 22, 2024 (Figure 1). Complete strategy is described in Supplement 1.

### Inclusion Criteria

*Inclusion criteria:* Articles in English assessing patients undergoing awake hand surgery (eg, carpal tunnel release, trigger finger, and Dupuytren's, fractures distal to the distal radius) with the use of an upper extremity pneumatic tourniquet (whereby studies used the term “pneumatic,” or described inflation of a tourniquet, including the pressure [mmHg]), and under regional or local anesthetic, without sedation.

### Exclusion Criteria

Studies were excluded if any of the following were met: (1) case reports, (2) nonprimary research studies, (3) reviews, (4) nonpeer reviewed studies (ie, dissertations), (5) procedures that were not hand surgeries (ie, proximal to the carpal bones, wrist or forearm surgery, other areas of the body), (6) pediatric patients, (7) the use of more than one tourniquet during the procedure on the same patient, (8) no specified tourniquet location, and (9) intravenous regional anesthetic or Bier block, IV sedation, or general anesthetic.

### Study Selection and Assessment

Two reviewers (KY and LK) independently screened the title and abstracts of articles retrieved from the primary search. Following the exclusion of duplicate studies and abstract screening for relevance, articles were screened at the full-text level by the same reviewers (KY and LK) who assessed them for eligibility independently based on the inclusion and exclusion criteria, and a third reviewer (JG) for any discrepancies. Further disagreements between the reviewers were resolved by a fourth reviewer (CL).

### Data Extraction

Specific variables were extracted from the selected articles. Based on our study objectives, these variables included: study information (title, author, country, date of publication, setting, institution name, aim of study, study design, study start and end dates, study length), participant information (number of patients, number of procedures done per patient, gender, age), procedure information (type of procedure, tourniquet location), and outcome information (tourniquet time, tourniquet tolerance; as reported by pain scale scores, overall patient satisfaction, any surgeon related outcomes such as rating of hemostasis, and postoperative complications). Two reviewers (KY and LK) extracted data from the included articles independently. Studies that included more than one tourniquet location (ie, comparison studies) were treated as individual studies.

## Results

**Figure 2.**
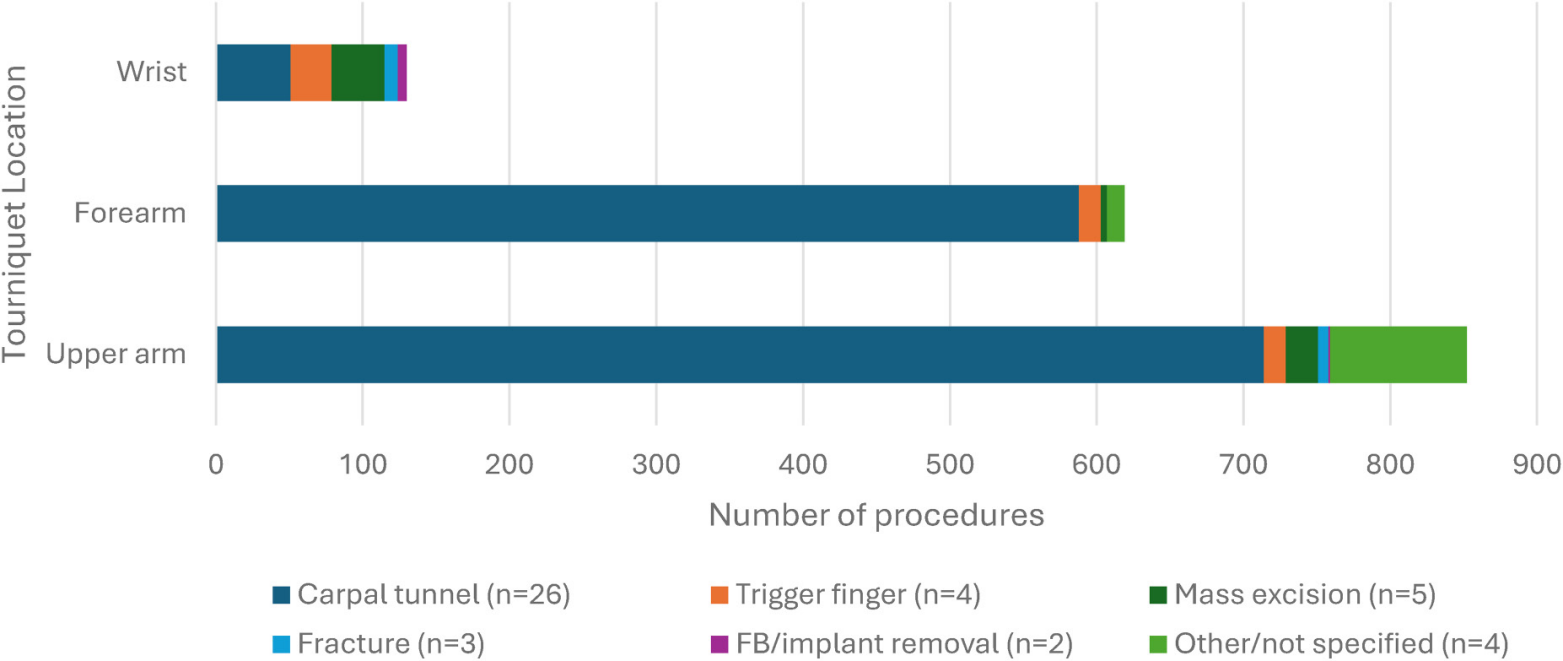
Proportion of procedures by tourniquet location.

**Figure 3.**
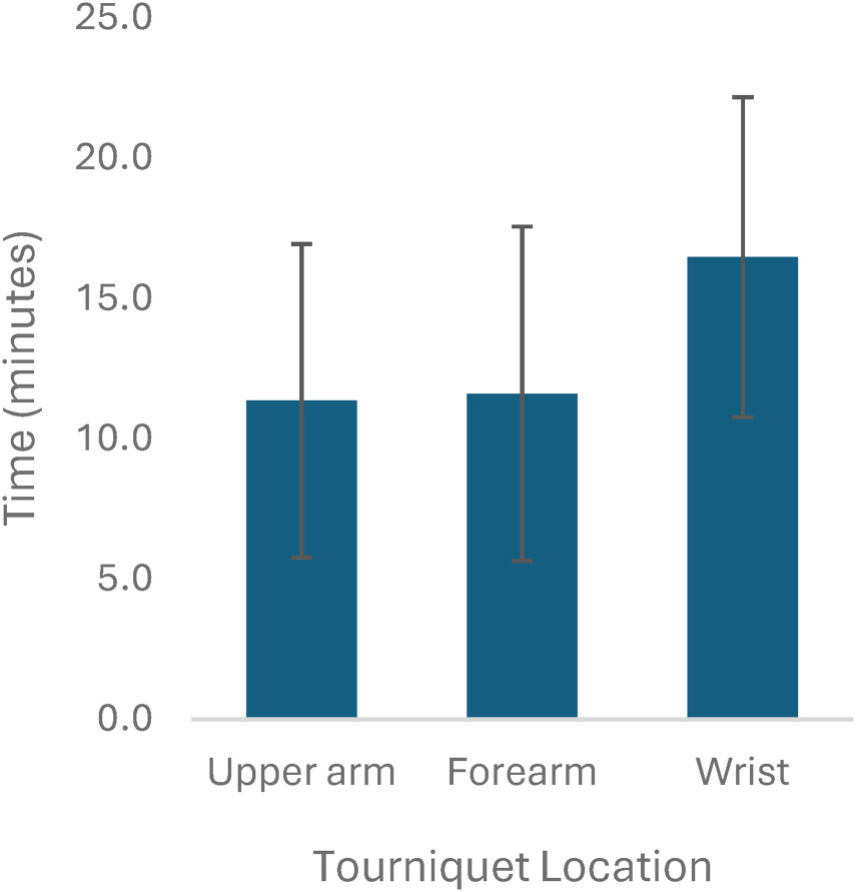
Average tourniquet time and standard deviations in minutes from 23 studies.

**Figure 4.**
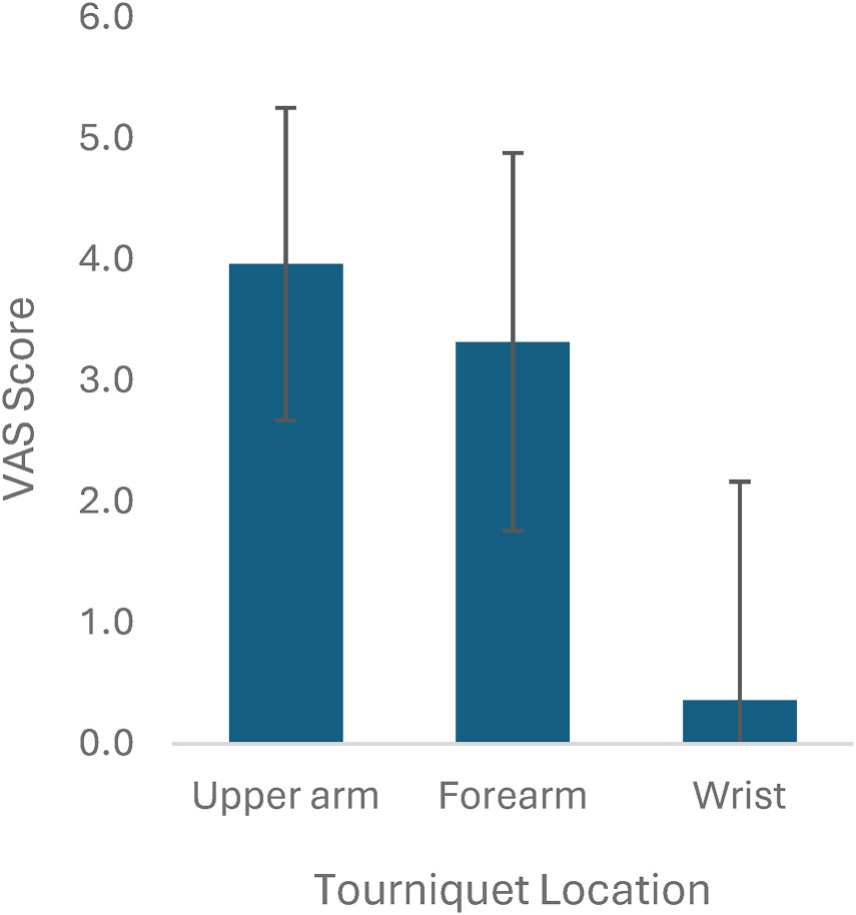
Tourniquet tolerance represented by average visual analog scale (VAS) scores from 22 studies.

Two hundred out of 1528 studies were reviewed at the full text level by 2 reviewers, and 26 studies were included.^2,6,8,9,12–33^ All 26 studies were done under local anesthetic. Of these 26 studies, there were 4 studies that included more than one tourniquet location (upper arm and forearm), and thus these were treated as individual studies, for a total of 30 studies for analysis. Overall, there were a total of 1670 participants and 1718 procedures. Of the 24 studies that reported patient gender and 23 studies that reported patient age, there were a total of 997 females and an average age of 55.7 years, respectively. Carpal tunnel release was the most common procedure (n = 1353, from 26 studies), followed by trigger finger release (n = 58, from 4 studies), other/not specified which included tendon repairs and finger amputations (n = 105, from 5 studies), mass/ganglion excisions (n = 62, from 5 studies), fractures (n = 16, from 3 studies), and foreign body/implant removal (n = 7, from 2 studies) (Figure 2). Studies were stratified by tourniquet location. There were 19 studies using upper arm tourniquets, 9 studies using forearm tourniquets, and 2 studies using wrist tourniquets. All studies used a pneumatic tourniquet. Mean tourniquet time across all studies was 11.9 minutes (2-55 minutes). Mean tourniquet time for upper arm, forearm, and wrist tourniquets was 11.4, 11.6, and 16.5 minutes, respectively (Figure 3). Mean visual analog score (VAS) for tourniquet pain across all studies was 3.4 out of 10. Mean VAS score for upper arm, forearm, and wrist tourniquets were 4.0, 3.3, and 0.4, respectively (Figure 4). Full results and extracted outcomes can be seen in Table 1.

**Table 1.** Demographic, Procedure, and Tourniquet Data from Studies Evaluating Tourniquet Location and Outcomes (n = 30).^a^

Tourniquet location	Studies (n = 30)	Procs (n = 30)	Patients (n = 30)	Females (n = 24)	Avg age (n = 23)	CTR (n = 26)	TFR (n = 4)	Mass excision (n = 5)	Fracture (n = 3)	FB/implant removal (n = 3)	Other (n = 5)	Avg TT (min) (n = 23)	Range TT (min) (n = 14)	VAS (n = 22)
Upper arm	19	931	906	463	55	714	15	22	7	1	93	11.4	2-55	4.0
Forearm	9	657	642	451	55	588	15	4	0	0	12	11.6	3-27	3.3
Wrist	2	130	122	83	43	51	28	36	9	6	0	16.5	5-29	0.4
All studies	30	1718	1670	997	55	1353	58	62	16	7	105	11.9	2-55	3.4

Abbreviations: Avg, average; CTR, carpal tunnel release; FB, foreign body; TFR, trigger finger release; TT, tourniquet time; VAS, visual analog scale.

^a^ Data from studies that included both upper arm and forearm tourniquets have been included in each upper arm and forearm category and treated as individual studies. The n in each column title represents the number of studies that provided this data.

### Wrist Tourniquets

The 2 wrist tourniquet studies^8,21^ included 122 patients (83 females, mean age 43) and 130 hand surgeries (51 carpal tunnel releases, 28 trigger finger/thumb releases, 36 mass excisions, 9 finger fractures, and 6 foreign body/implant removals). Average tourniquet time was 16.5 min (range 5-50 min), mean VAS score for tourniquet pain was 0.4 (range 0-10).

### Forearm Tourniquets

The 9 forearm tourniquet studies^2,6,9,13,17,18,28,32^ included 642 patients (451 females, mean age 54.6) and 657 hand surgeries (588 carpal tunnel releases from 8 studies, 15 trigger finger/thumb releases from 1 study, 4 mass excisions from 1 study, and 12 others from 1 study). Average tourniquet time was 11.6 min (range 3-27 min) from 6 studies, and mean VAS score for tourniquet pain was 3.3 (0-10) from 7 studies.

### Upper Arm Tourniquets

The 19 upper arm tourniquet studies^2,6,9,12,14–16,19,22–27,29–33^ included 906 patients (463 females, mean age 54.5) and 931 hand surgeries (714 carpal tunnel releases from 16 studies, 15 trigger finger/thumb releases from 1 study, 22 mass excisions from 2 studies, 7 fractures from 1 study, 1 foreign body removal from 1 study, and 93 others from 3 studies). Average tourniquet time was 11.4 min (range 2-55 min) from 14 studies, and mean VAS score for tourniquet pain was 4.0 (0-10) from 13 studies.

## Discussion

In this scoping review, we identified studies that used an upper extremity tourniquet (upper arm, forearm, or wrist) for awake hand surgery under regional or local anesthetic. The main outcomes from our review demonstrated that wrist tourniquets were the most tolerable, with the lowest VAS score by greater than 3 points, and had the longest tourniquet time (16.5 min) compared to forearm and upper arm tourniquets. The discrepancy in sample size and study designs made it challenging to compare across groups. However, our study illustrates that there is an inverse correlation between tourniquet time and VAS pain score. This aligns with the general hypothesis that wrist tourniquets are more comfortable and can be tolerated longer, likely as a reflection of less tissue around the wrist susceptible to ischemia/nerve palsies.

When comparing across all studies, wrist tourniquet studies had, on average, a tourniquet time of 4 min greater than the forearm and upper arm tourniquet studies. Carpal tunnel release was the most frequent procedure across all studies. The mean operative time for awake carpal tunnel releases using a tourniquet was reported to range from 6.9 to 37 min from 3 randomized control studies as reported in a systematic review and meta-analysis by Olaiya et al.^34^ This extrapolated observation shows that the average tourniquet time from the wrist tourniquet studies (as well as forearm and upper arm tourniquet studies) is all within the normal range of operative/tourniquet time as demonstrated in the literature. Furthermore, increased tourniquet time and better tolerance lends itself to flexibility for the type of procedure. For example, the tourniquet time may be less important for a straightforward carpal tunnel release if the lower end of tourniquet time is less than 10 min; however, a patient undergoing a more difficult procedure like a palmar fasciectomy for Dupuytren's disease may benefit from a wrist tourniquet as the procedure itself may take over 30 min. Overall, the increased tourniquet time could be due to multiple factors, including more operative flexibility for the surgeon in terms of time management with less concern for patient pain, or surgeon expertise and experience. Our findings of lower VAS with longer tourniquet times with wrist tourniquet contradicts the published literature indicating that increased tourniquet duration correlates with increased pain.^32^

Our review demonstrated that wrist tourniquets were the most comfortable, followed by forearm, then upper arm tourniquets, as represented by VAS scores. Multiple studies, including one by Maury et al,^35^ reported that distal tourniquets are better tolerated than upper arm tourniquets, aligning with this study's results. The VAS is the most used pain scale due to feasibility for both the clinician and patient. However, only 68% (n = 21/31) of the included studies used the VAS. The 10 remaining studies either used a subjective measure (eg, recorded number of patients reporting none, minimal, moderate, or severe pain) or did not use any measurement instrument. We also aimed to look at surgeon-related outcomes, including adequate hemostasis (eg, bleeding intraoperatively), and postoperative complications. However, these outcomes were either not measured or not well documented by the included studies. When assessing the effect of tourniquets, both patient-reported and surgeon-reported outcomes should be measured, or core outcome sets should be utilized. Core outcome sets are beneficial as they can enhance the relevance of research by ensuring researchers are measuring standardized outcomes across a field.^36^ Currently, there are no core outcome sets for upper extremity tourniquet use in hand surgery. A recent systematic review by Levit et al^37^ compared wide awake local anesthetic no tourniquet to local anesthetic with tourniquet for trigger finger releases. They reported on 8 outcomes they deemed to be critical, including patient pain, patient satisfaction, physician satisfaction, analgesic use, functional outcomes, adverse events, procedure time, and costs. These outcomes may be applicable as trigger finger releases can be done with a wrist tourniquet. Overall, core outcome sets may assist in standardizing outcomes in future hand research, and for our purpose, comparing different upper extremity tourniquets.

All 3 tourniquet locations (upper arm, forearm, and wrist) effectively achieve a bloodless surgical field. While wrist tourniquets have been shown to be more comfortable for patients in some studies, the choice of tourniquet location ultimately depends on balancing patient comfort with the practical requirements of the specific surgical procedure being performed. Surgeons must consider these factors to ensure optimal surgical conditions while minimizing patient discomfort and potential complications. A wrist tourniquet is often sufficient and preferred method for standard hand procedures. It allows for good visualization of the surgical site while minimizing the amount of upper extremity tissue under tourniquet pressure.^10^ However, if during the procedure the surgeon determines that a more proximal release is necessary, the wrist tourniquet would no longer be adequate. This underscores the importance of surgical flexibility and the need to balance optimal surgical conditions with patient comfort and safety.

The findings of this scoping review must be interpreted within the context of its limitations. First, it is possible that despite being rigorously developed, we may have missed relevant citations during our search as it was limited to the English language. As this was a scoping review, by design, the methodology provided a broad overview rather than an in-depth analysis on the current literature on upper extremity tourniquets, which can result in generalized findings that require further detailed examination. Second, scoping reviews typically do not include a critical appraisal or assessment of the quality and risk of bias of the included studies, a limitation noted in 16% of scoping reviews.^38^ As our inclusion criteria required patients to be undergoing hand surgery, studies conducted with volunteer participants were excluded, although these studies may also provide objective outcomes comparing tourniquets on the upper arm, forearm, and wrist.^39^ However, as described in the recently published PRISMA extension for scoping reviews, article quality assessment is not a typical feature of scoping reviews unless it aligns with the objectives of the review and is practical to complete.^40^ Finally, while scoping reviews effectively map available evidence, they generally do not synthesize findings to the same extent as systematic reviews. As demonstrated, there were limited outcomes for each tourniquet location, as not all studies reported outcomes. The exploratory nature of scoping reviews makes them less suitable for addressing very specific research questions.

We reviewed the use of upper extremity tourniquets for hand surgery and their associated outcomes. While limited (n = 2), the literature suggests that wrist tourniquets are the most comfortable tourniquet location and are well tolerated by both patients and surgeons. While this study effectively mapped the current evidence on wrist tourniquets for hand surgery, more robust clinical studies need to be conducted using wrist tourniquets, with a clear definition of what constitutes a wrist tourniquet. While wrist tourniquets show promising outcomes, especially in terms of comfort, there is still a need for more comprehensive research on their impact on surgical efficacy and surgeon preference. Future research should include prospective and comparative clinical studies.

## Supplemental Material

sj-docx-1-psg-10.1177_22925503251392574 - Supplemental material for The Use of Upper Extremity Tourniquets in Hand Surgery—Does Tourniquet Location Make a Difference? A Scoping Review Supplemental material, sj-docx-1-psg-10.1177_22925503251392574 for The Use of Upper Extremity Tourniquets in Hand Surgery—Does Tourniquet Location Make a Difference? A Scoping Review by Kimberley Yuen, Laryssa Kemp, Isabella Churchill, Jessica Gormley, Cameron Leveille, Helene Retrouvey, Matthew McRae and Ammara Ghumman in Plastic Surgery
