# Supplementary figures and images for: The Use of Upper Extremity Tourniquets in Hand Surgery—Does Tourniquet Location Make a Difference? A Scoping Review
Source: Plast Surg (Oakv). 2025 Dec 9:22925503251392574. Online ahead of print. doi: 10.1177/22925503251392574 (PMC12689351; doi:10.1177/22925503251392574)

**Supplement**
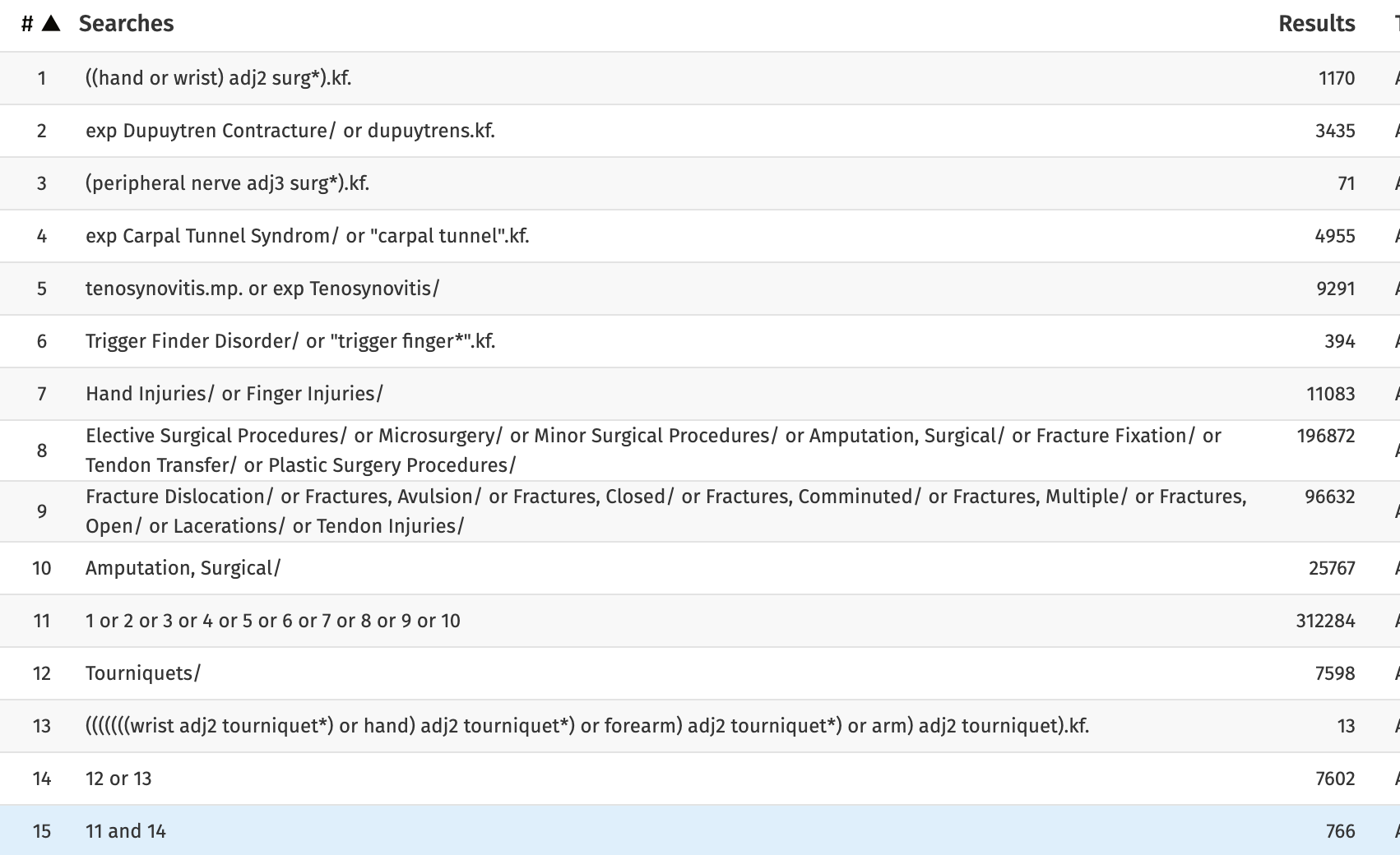

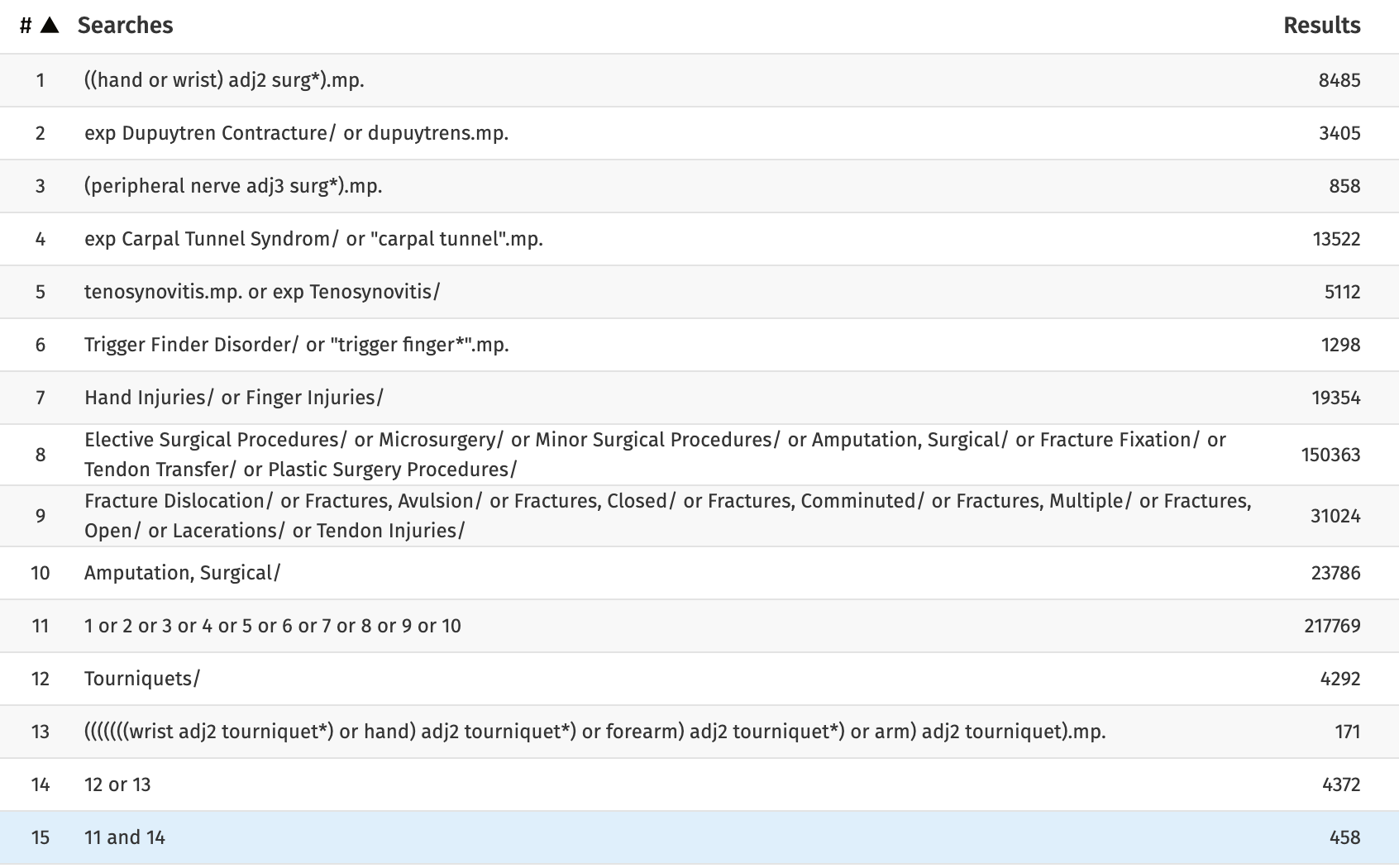


Supplement 1. Search strategies for OVID Medline and Embase databases

Supplement: sj-docx-1-psg-10.1177_22925503251392574 - Supplemental material for The Use of Upper Extremity Tourniquets in Hand Surgery—Does Tourniquet Location Make a Difference? A Scoping Review [file sj-docx-1-psg-10.1177_22925503251392574.docx]
